# Basal and post-ischemic vascular compliance in children/adolescents born small for gestational age

**DOI:** 10.1007/s00467-012-2168-y

**Published:** 2012-05-10

**Authors:** Mirella Strambi, Gianluca Messa, Silvia Berni, Serena Capitani, Andrea Pammolli, Francesca Iacoponi, Costantina Censurato, Caroline Magne Tene, Alessandro Fiorica, Aurelio Vittoria

**Affiliations:** 1 Department of Paediatrics, Obstetrics and Reproductive Medicine, University of Siena, Viale Bracci 36, 53100 Siena, Italy; 2 Department of Clinical Medicine and Immunological Science, University of Siena, Siena, Italy; 3 Department of Physiopathology, Experimental Medicine and Public Health, University of Siena, Siena, Italy; 4 Department of Biomedical Sciences, Section of Applied Biology, University of Siena, Siena, Italy

**Keywords:** Hypertension, Small for gestational age, Vascular disease, Arterial stiffness

## Abstract

**Background:**

Intrauterine growth restriction plays a powerful role in influencing later susceptibility to certain chronic diseases, such as hypertension. Endothelial dysfunction and arterial stiffness are early events in the development of cardiovascular diseases (CVDs). We have studied vascular compliance in small for gestational age (SGA) children/adolescents in comparison with that in appropriate for gestational age (AGA) subjects.

**Methods:**

We monitored blood pressure, vascular resistance and compliance in 82 children–adolescents (52 SGA, 30 AGA), by means of pulse wave analysis (CR 2000 HDI) at the radial level, before and after 3 min of ischemic stress at the brachial level.

**Results:**

In the children/adolescents born SGA we found a significant increase in systolic and diastolic blood pressure and vascular resistance in the basal condition; the large and small vessels were stiffer. After ischemia we observed an increased vascular response in the SGA children/adolescents: there was a great diminution of systolic and diastolic blood pressure and a larger increase of the elasticity of the conduit and resistance vessels.

**Conclusions:**

These data show that the SGA group presented some early signs of arterial wall functional disorders. More pediatric data are needed for the evaluation by non-invasive techniques of vascular function in children–adolescents at risk of CVD.

## Introduction

An extensive body of research has shown that birth weight is related to adverse outcomes, such as hypertension, risk of coronary heart disease and type 2 diabetes, with smaller babies having a greater risk with age [1–4], with findings of the early studies of Barker et al. [1, 2] being confirmed in later studies [3, 4]. An inverse relationship between adult blood pressure and gestational age has been found in both adult men and women who were born prematurely [5, 6] and repeatedly confirmed [7, 8]. In the context of a low birth weight (LBW) considered to be an expression of an increased risk of developing cardiovascular diseases (CVDs) later in life, recent studies have indicated that it may not be preterm birth per se that is a risk factor for CVDs but rather the relationship between intrauterine growth restriction (IUGR) and cardiovascular or metabolic disorders in adult life [9–12]. The underlying mechanisms are still not clear, but genetic factors associated with fetal growth restriction and increased risk of CVDs may be involved [13–15].

Reduced arterial elasticity, measured also in terms of vascular compliance, stiffness, or distensibility, is an early sign of vascular damage and can be used as a surrogate marker of arterial function [16, 17]. Assessment of arterial elastic properties helps to identify subclinical cardiovascular pathologies [18], enabling prevention and targeted early treatment [16], and it may also enable the effects of therapy to be evaluated [19, 20].

Vascular endothelial dysfunction is a key event in the development of diseases associated with birth weight [21], and a positive association between LBW and endothelial dysfunction has been demonstrated in young adults [22, 23] and children [21, 24]. The evaluation of brachial artery endothelial function (flow-mediated dilation, FMD) is now increasingly used for paediatric cardiovascular risk evaluation [25].

To explore vascular function in a very early phase of life (children or adolescents) we studied the vascular compliance of two groups of subjects divided according to birth weight. We checked arterial compliance before and after ischemic stimuli in a group of SMA children/adolescents monitoring conduit compliance and vessel resistance by radial pulse wave analysis. We also examined induced vascular reactivity after a standardized ischemic stimulus.

## Methods

### Study population

From April 2008 to April 2009, we studied 82 children and adolescents (48 boys, 34 girls, age range 8–16 years). They were divided into two groups according to birth weight and gestational age [26, 27]. IUGR, antenatally diagnosed, was defined as insufficient fetal growth <2 standard deviation (SD) (or <3rd percentile) below the average for gestational age, and SGA at birth was defined as insufficient body size of <2 SD (or <3rd percentile) below the average for weight and/or length in relation to gestational age and gender for the Italian population.

We enrolled 52 Italian children/adolescents with a gestational age of 37–38 weeks into the SGA/IUGR group (32 males, 20 females); these children had been followed up in a regular clinical program. The average birth weight was 1,684 ± 520 g, the average head circumference at birth was 31.8 ± 1.8 cm, and the average age at the time of examination was 12.40 ± 2.47 years. The control group comprised 30 children/adolescents (16 males, 14 females) born full term and with the appropriate weight for gestational age (AGA); the average age at the time of examination was 12.27 ± 1.74 years. The subjects of both groups were nonobese, and they were all at Tanner developmental stage 2.

Blood pressure measurements were obtained from the right arm of each subject in a supine position. The proper cuff size was ensured by arm measurements. Systolic and diastolic blood pressures (SBP, DBP, respectively) were determined by automated oscillometric monitors (HDI/Pulse Wave CR-2000; Hypertension Diagnostics, Eagan, MN). The clinical characteristics of the 52 SGA children/adolescents and the 30 age- and sex-matched AGA children/adolescents of the control group are summarized in Table 1.

**Table 1:** Demographic characteristics of patients of the two groups

Demographic characteristics	AGA (*n* = 30)	SGA (*n* = 52)	*P* (*t* test)
Gender (M/F)	16/14	32/20	0.621
Age (years)	12.27 (1.74)	12.40 (2.47)	0.800
Weight (kg)	46.42 (9.61)	48.67 (14.42)	0.449
Height (cm)	153.21 (10.26)	153.93 (16.14)	0.827
Body size	1.41 (0.17)	1.40 (0.29)	0.864
BMI (kg/m^2^)	19.69 (3.03)	20.86 (4.39)	0.200

AGA, appropriate weight for gestational age (normal birth weight); SGA, small for gestational age (low birth weight); M, male; F, female; BMI, body mass index

Exclusion criteria were known congenital or acquired cardiovascular, hepatic, renal, or brain diseases, genetic syndromes, and chronic gastrointestinal diseases, such as celiac, metabolic, or endocrine disorders.

The Institutional Local Ethical Committee of the Siena University Hospital (AOUS S. Maria alle Scotte) approved the study. Informed consent was obtained from older children and from both parents of all children after a full explanation of the study prior to enrolment.

### Study protocol

The subjects were studied between 1500 hours and 1800 hours; after 15 min of rest in a quiet, temperature-controlled room, with the subject in a recumbent position, BP measurement and pulse wave analysis (PWA) were performed three times at 5-min intervals. We then provoked an ischemic stimulus with a cuff placed on the brachial segment of the forearm inflated to a pressure exceeding the systolic arterial blood pressure so that the radial homolateral oscillometric curve disappeared for exactly 3 min. The BP measurement and PWA were repeated four times at 5-min intervals.

### Assessment of arterial elasticity

Diastolic pulse contour analysis uses a modified Windkessel model to derive information on proximal and distal arteries by analyzing the diastolic portion of the pressure pulse contour; we measured the arterial waveform in the nondominant arm with a cardiovascular profiling instrument (HDI/Pulse Wave CR-2000; Hypertension Diagnostics). Briefly, the tonometer was applied to the patient’s radial artery at the wrist overlying the radial bony prominence. The subject’s arm was supported by a wrist stabilizer for optimal positioning and minimal movement during the measurements. The cuff for BP measurement was placed on the contralateral arm and inflated concurrently with the pulse waveform recording for calibration; a 30-s interval of analogue waveforms was digitized at 200 samples/s, and a beat marking algorithm was determined the beginning of systole, peak systole, onset of diastole, and end of diastole for all beats in the 30-s measurement period. The elasticity indices of the arteries (C1 and C2) were quantified during the diastolic portion of the heart cycle (mean recording time 30 s). According to the modified Windkessel model of circulation, C1 is a marker of large artery elasticity and C2 is a marker of small artery elasticity. Heart rate, mean arterial pressure (MAP), and stroke volume were also calculated from the radial pressure waveform using HDI/Pulse Wave CR-2000 software. These hemodynamic parameters (i.e. MAP and stroke volume) are used in multivariate algorithms for determining C1 and C2. The full method has already been validated and described in detail in adult patients [18]; however, they have not yet been validated in the pediatric population [28].

### Statistical analysis

All variables were tested for normal distribution by the Kolmogorov–Smirnov test and for variance homogeneity by the Levene test. The variables are expressed as average values and SD or as absolute frequency.

The AGA and SGA groups were compared under basal conditions using the unpaired *t* test or proportions test. The one-way analysis of variance, with the Dunn post hoc test for multiple comparisons when necessary, was used to compare the determinations of each group (AGA and SGA). A* P* value of <0.05 was considered to be statistically significant. The statistical analysis was performed using SPSS statistical software ver. 14.0 (SPSS, Chicago, IL).

## Results

### Subjects and vascular function

A total of 82 children/adolescents were studied: 30 AGA (16 males, 14 females) and 52 SGA (32 males, 20 females). Table 1 summarizes the demographic characteristics of all subjects at the time of the study. There were no significant differences between the two groups in terms of demographic variables, with the children/adolescents in the AGA and SGA groups being homogeneous for gender distribution, age, weight, height, body size, and body mass index (BMI).

With respect to vascular function and arterial elasticity, the comparison between the two groups at basal time revealed significant differences between the AGA and SGA groups in SBP (*t* test,* P* < 0.01, with a difference of + 7.6 mm Hg) and DBP (*t* test,* P* < 0.05, with a difference of + 4.23 mm Hg); significant negative differences were highlighted in C1 (*t* test,* P* < 0.01, with a difference of −2.21 ml/mmHg × 10) and in C2 (*t* test, *P* < 0.01 with a difference of −1.53 ml/mmHg × 100). There were also significant difference in vascular resistances (VR) (*t* test,* P* < 0.05 with +151 dyne/s × cm^-5^). However, there was no statistical difference between the two groups for the pulse pressure (PP) variable (*t* test,* P* = 0.177). (Table 2)

**Table 2:** Vascular compliance in the appropriate for gestational age (AGA) group

Vascular variables^a^	Basal	P-I_1_	P-I_2_	P-I_3_	P-I_4_
SBP	111.1 (10.3)**	109.4 (9.3)	111.2 (9.92)	109 (7.66)	111.6 (9.84)
DBP	54.8 (6.1)	54.4 (6.03)	54.4 (7.66)	51.4 (9.81)	53.5 (6.75)
PP	56.1 (6.3)**	53.6 (5.42)	53.73 (6.95)	56.33 (9.05)	54.6 (5.51)
C_1_	12.89 (4.07)*	12.83 (4.02)	13.24 (5.78)	12.82 (4.24)	12.66 (3.31)
C_2_	7.78 (2.7)**	8.13 (2.98)	7.65 (1.42)	7.50 (1.79)	7.22 (2.07)
VR	1184 (230)	1068 (346)	1157 (186)	1105 (174)	1166 (213)

*P = 0.04; **P = 0.002, basal values versus SGA

Values are given as the mean with the standard deviation (SD) in parenthesis

Basal, Mean of three consecutive determinations, each of 5-min duration; P-I_1,2,3,4_, post-ischemic determination, each of 5-min duration

^a^SBP, Systolic blood pressure (mmHg); DBP, diastolic blood pressure (mmHg); PP, pulse pressure (mmHg); C_1_, macrovascular compliance (ml/mm Hg × 10); C_2_, microvascular compliance (ml/mm Hg × 100); VR, vascular resistance (dyne/s × cm^-5^), before and after brachial ischemia (3’)

### Post-ischemia vascular function

The changes induced by the ischemic stress were analyzed separately in each group, AGA and SGA, at standardized times, followed by an analysis of the changes between the two groups.

After the standardized ischemic stress had been applied to the brachial artery, we noticed a modest but not significant decrease of SBP (*P* = 0.349), DBP (*P* = 0.149), and pulse pressure (PP) (*P* = 0.191) in the AGA group. The multiple comparisons in the AGA group showed a significant difference for the variables C1 (*P* = 0.949), C2 (*P* = 0.348), and vascular resistance (*P* = 0.949). In the SGA group, the change in SBP (*P* = 0.096), DBP (*P* = 0.444), PP (*P* = 0.075), and C2 (*P* = 0.105) following application of the ischemic stimulus was not significant; however, there was a significant increase of C1 (*P* < 0.01) and a significant reduction of vascular resistance (VR) (*P *< 0.001). In particular, we found a significant increase (+1.73 mmHg) between basal time and the last time for C1 (Dunn post hoc, *P* < 0.05), and a significant reduction between basal time and each time of post-ischemic determination for VR (−117 dyne/s × cm^-5^) (Table 3).

**Table 3:** Vascular compliance in the SGA group

Vascular variables^a^	Basal	P-I_1_	P-I_2_	P-I_3_	P-I_4_
SBP	118.7 (11.2)**	115.8 (12.2)	116.6 (12.1)	116.3 (10.6)	114.6 (11.05)§
DBP	59.03 (7.6)	59.6 (9.86)	58.1 (9.11)	57.5 (7.27)	59.1 (6.73)
PP	58.4 (7.9)**	56.1 (10.1)	57.5 (9.17)	57.3 (9.5)	55.54 (7.84)§§
C_1_	10.68 (3.37)*	12.21 (4.57)	10.96 (5.23)	11.94 (4.22)	12.41 (4.57)§§
C_2_	6.25 (2.0)**	6.52 (2.46)	6.27 (2.43)	6.37 (2.68)	7.07 (2.82)§§
VR	1335 (350)	1256 (263)	1242 (254)	1218 (196)	1228 (224)§§

*P < 0.04; ** *P* < 0.002, basal values vs. AGA

§*P* < a 0.02; §§*P *< 0.002, basal values vs. post-ischemic modifications

Values are given as the mean with the SD in parenthesis

SGA, small for gestational age; AGA, appropriate for gestational age; SBP, systolic blood pressure; DBP, diastolic blood pressure; PP, pulse pressure; C_1_, macrovascular compliance (ml/mm Hg × 10); C_2_, microvascular compliance (ml/mm Hg × 100); VR, vascular resistance

**Fig. 1:**
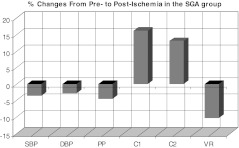
Changes (%) in systolic (*SBP*) (*P* < 0.02) and diastolic blood pressure (*DBP*) (*P* = not significant), pulse pressure (*PP*) (*P* < 0.02), macrovascular compliance (*C1*) (*P* < 0.002), microvascular compliance (*C2*) (*P* < 0.002), vascular resistance (*VR*) (*P* < 0.002) in children/adolescents born small for gestational age (*SGA*) from pre- to post-ischemia

Our analysis of post-ischemic vascular compliance in both groups revealed a vasodilatory response, which did not reach statistical significance in the AGA. In contrast, a more lively vasodilator response was observed in the SGA group, which was characterized by a greater decrease in SBPc (−4.1 mm Hg in the SGA group vs. −2.1 mm Hg in the AGA group), DBP (−1.57 vs. –1.3 mm Hg, respectively) and VR (−117 in SGA vs. −116 in AGA). We also observed a greater increase in the indexes of macrovascular compliance [+1.73 vs. +0.4 (ml/mm Hg × 10) in SGA vs. AGA groups] and microvascular elasticity [+0.82 vs. +0.35 (in ml/mm Hg × 100) in SGA vs. AGA groups]. The percentage changes from pre- to post-ischemia in the SGA group are shown in Fig. 1.

## Discussion

Knowledge of the characteristics of the vascular tree in IUGR infants, children, and young adults can help clarify the underlying mechanisms that are at the origin of hypertension in this population at increased risk of CVD in adulthood.

In the SGA group we observed an increase in systolic, pulse, and diastolic blood pressure and in vascular resistances; the elasticity of the large and small vessels was lower in the SGA than in the AGA group. These data are in line with those reported by other authors [1, 26–29] who found an increase in SBP in many epidemiological studies, suggesting an inverse relationship between birth weight and hypertension. The increase in these hemodynamic parameters, associated with significantly worse vascular compliance in the SGA group, could indicate that in this group of selected healthy children/adolescents there are very early signs of functional impairment of the arterial wall, which in turn depends on the function of endothelial cells. The endothelium controls permeability, regulates vascular growth, and keeps the blood vessels open and free from clots by releasing a number of vasoactive factors, such as nitric oxide (NO), angiotensin II, endothelin, among others. Endothelial impairment, when identified, is an early risk factor of coronary or peripheral artery disease. Arterial stiffness also seems to have a genetic component that is largely independent of the influence of blood pressure and other cardiovascular risk factors [30–35]

When we provoked a standardized ischemic stress at the brachial artery of subjects in the AGA group, we observed a decrease in the SBP (−2.1 mm Hg), DBP (−3.4 mm Hg), PP (−2.5 mm Hg), and VR (−111 dyne/s × cm^−5^) and an increase in the C1 (+0.35 ml/mm Hg × 10) and C2 (+0.35 ml/mm Hg × 100). Following the same stress in the SGA group, we observed a more lively vasodilator response that was characterized by a greater decrease in SBP (−4.1 mm Hg), DBP (−1.53 mm Hg), PP (−2.86 mm Hg), and VR (−117 dyne/s × cm^−5^). We also observed a greater increase in the indexes of macrovascular compliance (C1 + 1.73 ml/mm Hg × 10) and microvascular elasticity (C2 + 0.82 ml/mm Hg × 100). These data support the hypothesis that in the SGA group the response induced by ischemic stress is greater, which could indicate that endothelial cells are chronically overstimulated to supply organs and tissues. This pathophysiological condition could not only increase oxidative stress on the vascular tree, thereby favoring progressive early damage to the vascular wall, for example by increasing stiffness in large and small arteries, but it could also explain the early appearance of hypertensive disease with the manifestation of endothelial dysfunction due to decreased synthesis and release of NO and other vasorelaxant substances. Mechanical forces, comprising both unidirectional laminar and oscillatory shear, are increasingly being recognized as important inducers of vascular NO and generators of reactive oxidative species (ROS). On the other hand, oscillatory shear is linked to increased ROS production with consequent oxidative damage, as occurs in hypertension. As recently reviewed, shear stress alone potently stimulates the release of vasorelaxant mediators, such as NO, enhances endothelial survival, and counters cell adhesion and thrombosis [36].

Our study has a number of limitations. First, the two experimental groups consisted of a relatively small number of children and adolescents matched for age, but the match for gender and height was not perfect. Secondly, experience with applanation tonometry and other technologies (Doppler ultrasound, photoplethysmography, among others) in pediatric patients is limited; methods such as Doppler ultrasound are strictly dependent on highly trained personnel, and the few studies performed to date have involved relatively few subjects [28, 37]. However, such equipment has a relatively low cost, is easy to use, and is acceptable to patients; in addition, such equipment provides important information on the vascular wall and flow-mediated dilation.

In conclusion, we found a raised SBP and a reduced vascular elasticity under basal conditions in SGA children/adolescents; after the application of ischemic stress, the large and small vessels of these children/adolescents manifested a stronger vasodilation than those of AGA subjects. However, further studies with more subjects are required so that the prognostic value, relative to established predictors of risk, may be fully defined, especially in the pediatric population. Moreover we want to stress that the applanation tonometry method is absolutely not invasive and would appear to be particularly suitable for the pediatric/adolescent patient population, for which we need to have simple techniques that are non-invasive so as to increase the compliance of families and healthy children/adolescents to prevent cardiovascular disease [38].
